# Computer-Aided Screening of Phytoconstituents from *Ocimum tenuiflorum* against Diabetes Mellitus Targeting DPP4 Inhibition: A Combination of Molecular Docking, Molecular Dynamics, and Pharmacokinetics Approaches

**DOI:** 10.3390/molecules27165133

**Published:** 2022-08-12

**Authors:** Harshit Sajal, Shashank M. Patil, Ranjith Raj, Abdullah M. Shbeer, Mohammed Ageel, Ramith Ramu

**Affiliations:** 1 Department of Biotechnology and Bioinformatics, JSS Academy of Higher Education and Research, Mysuru 570015, India; 2 Department of Pharmacology, JSS Medical College, JSS Academy of Higher Education and Research, Mysuru 570015, India; 3 Department of Surgery, Faculty of Medicine, Jazan University, Jazan 45142, Saudi Arabia

**Keywords:** DPP4 inhibition, *Ocimum tenuiflorum*, in silico approach, molecular docking, molecular dynamics simulations, binding free energy calculations, 1S-α-pinene, β-pinene, dehydro-*p*-cymene

## Abstract

Diabetes mellitus is a major global health concern in the current scenario which is chiefly characterized by the rise in blood sugar levels or hyperglycemia. In the context, DPP4 enzyme plays a critical role in glucose homeostasis. DPP4 targets and inactivates incretin hormones such as glucagon-like peptide-1 (GLP-1) and gastric inhibitory polypeptide (GIP) as physiological substrates, which are essential to regulate the amount of insulin that is secreted after eating. Since the inactivation of incretins occurs, the hyperglycemic conditions continue to rise, and result in adverse physiological conditions linked with diabetes mellitus. Hence, inhibition of DPP4 has been the center of focus in the present antidiabetic studies. Although few DPP4 inhibitor drugs, such as alogliptin, saxagliptin, linagliptin, and sitagliptin, are available, their adverse effects on human metabolism are undeniable. Therefore, it becomes essential for the phytochemical intervention of the disease using computational methods prior to performing in vitro and in vivo studies. In this regard, we used an in-silico approach involving molecular docking, molecular dynamics simulations, and binding free energy calculations to investigate the inhibitory potential of *Ocimum tenuiflorum* phytocompounds against DPP4. In this regard, three phytocompounds (1S-α-pinene, β-pinene, and dehydro-*p*-cymene) from *O. tenuiflorum* have been discovered as the potential inhibitors of the DPP4 protein. To summarize, from our in-silico experiment outcomes, we propose dehydro-*p*-cymene as the potential lead inhibitor of DPP4 protein, thereby discovering new a phytocompound for the effective management of hyperglycemia and diabetes mellitus. The reported compound can be taken for in vitro and in vivo analyses in near future.

## 1. Introduction

Type 2 diabetes mellitus (T2DM) is a chronic metabolic disorder associated with beta-cell dysfunction which emanates impaired insulin secretion, resistance to peripheral actions of insulin, or both, producing interminable hyperglycemia. Synergistically, with other metabolic aberrations, chronic hyperglycemia leads to the development of disabling and life-threatening health complications such as neuropathy, retinopathy, nephropathy, and cardiovascular diseases [1]. Despite the availability of different classes of oral medications and several injectable medications, glycemic control in diabetic patients remains unsatisfactory. The current scenario entails the recurrent discovery of new therapeutic targets and medicaments to mitigate T2DM, targeting various proteins involved in diabetic pathogenesis, through the discovery of small molecules as their inhibitors or activators [2].

The dipeptidyl peptidase 4 (DPP4), in particular, plays a crucial role in glucose homeostasis since it is responsible for inactivating incretins. DPP4 is a type II membrane protein with a widespread distribution in numerous tissues including the intestine, kidney, vascular endothelium, the liver and pancreas, glandular epithelial cells, and cells of the immune system (as the T-cell differentiation antigen, CD26) [3]. The DPP4 usually forms a homodimer on the cell surface for proteolytic activity. However, DPP4 also circulates in the plasma carrying catalytic activity even after shedding; besides, its higher level is associated with many diseases including diabetes [4]. As a serine exopeptidase, DPP4 cleaves dozens of peptides including neuropeptides, chemokines, incretins, and regulatory peptides, which chiefly consist of a proline or alanine residue at position 2 of the amino-terminal region. For instance, the incretin hormones, glucagon-like peptide-1 (GLP-1), and gastric inhibitory polypeptide (GIP) are targeted by DPP4, since they act as the physiological substrates of DPP4 [5].

Both GLP-1 and GIP manifest insulinotropic activity and account for around 50% of insulin secretion after meal intake. GLP-1 decreases glucagon secretion from alpha cells and plasma glucose levels. It acts as a regulator of satiety and appetite through the hypothalamus and improves blood sugar excursion, delaying food absorption by inhibiting gastric emptying [6], whereas GIP enhances insulin secretion via the GIP receptor (GIPR) on beta cells. As GIPR is also expressed in alpha cells, GIP contributes to insulin secretion by invoking paracrine alpha to beta cell communication [7]. DPP4 cleavage eliminates the classical glucoregulatory actions of GLP-1 and GIP. DPP4 targets and cleaves the receptor binding domain of GLP-1 and GIP, which results in the generation of peptide(s) with 100-fold less receptor affinity. This results in the elevation of blood sugar and associated effects of hyperglycemia. Therefore, inhibition of DPP-4 exerts an antihyperglycemic effect by preventing degradation of endogenously released GLP-1 and GIP levels, improved pancreatic islet responses (increased insulin and suppressed glucagon levels), as well as improved glucose homeostasis [5].

Oral hypoglycemic drugs are beneficial for the treatment of type II diabetes mellitus. In the current scenario, sitagliptin, saxagliptin, linagliptin, and alogliptin have been approved as novel gliptins by the Food and Drug Administration (FDA) to treat T2DM, whereas vildagliptin has been validated by the European Medicines Agency (EMA) [8]. However, these gliptins are associated with many adverse effects, including hypoglycemia (in conjunction with sulfonylureas) and have weight-neutral effects [9]. Sitagliptin and saxagliptin are associated with upper respiratory tract infection, nasopharyngitis, headache, arthralgia, urinary tract infection [10]. Hypersensitivity reactions such as anaphylaxis and angioedema are also reported with the prescribing information of most DPP-4 inhibitors [11]. Moreover, post marketing reports show that sitagliptin, vildagliptin, and saxagliptin are associated with acute pancreatitis, including fatal and non-fatal hemorrhagic or necrotizing variants [11,12].

Among the scientific community, the use of plants or herbal sources is becoming increasingly popular for the discovery and development of new anti-diabetic drugs that may control diabetes with the least unwanted side effects of conventional drugs. Several India originated medical plants have been proven to have effective antidiabetic activity. *Ocimum tenuiflorum* (*Tulsi*) is an aromatic perennial plant in the family *Lamiaceae*. It is native to the Indian subcontinent and is widely cultivated throughout Southeast Asia’s tropics. In Ayurveda, it aids in the treatment of cough, asthma, diarrhea, fever, dysentery, arthritis, eye diseases, indigestion, gastric ailments, etc. A number of phytochemical and pharmacological properties of *Tulsi* have been observed, including its anti-diabetic, antioxidant, wound healing, radiation protective, immunomodulatory, antistress, antifertility, anti-inflammatory, antimicrobial, and anticancer properties, highlighting the phytochemical profiling [13].

However, significant antidiabetic activity needs to be investigated on a variety of levels. Nowadays, in silico pharmacology methodologies are a vital part of the drug discovery process. Meanwhile, in order to strategically organize our biological investigations, we wish to evaluate the antidiabetic potential of *O. tenuiflorum* phytocompounds using bioinformatics tools. In the field of drug discovery, computational techniques including molecular docking, molecular dynamics simulation, and calculations of binding free energies have been shown to produce accurate predictions. When compared to in vitro and in vivo investigations, they end up saving a significant amount of time and money. Therefore, we aim to virtually screen the *Tulsi* phytochemicals obtained from the Indian Medicinal Plants, Phytochemistry, and Therapeutics (IMPPAT) database as the potential inhibitors of the DPP4 protein, through molecular docking simulation, molecular dynamics (MD) simulation, binding free energy calculation, and pharmacokinetic analysis. The outcomes of this study proffer the *Tulsi* phytochemicals as the potent site-specific inhibitors of DPP4, which can be considered for biological evaluation in the upcoming days.

## 2. Materials and Methods

### 2.1. Molecular Docking Simulation

The X-ray crystal structure of human dipeptidyl-peptidase 4 or DPP4 bound with N7F (PDB ID: 4A5S; chain A) was retrieved from the RCSB PDB database. The length of the retrieved protein structure was 728 residues, expanding from Arg40 to Ala767, whereas the structures of 61 available ligand molecules were retrieved from the Indian Medicinal Plants, Phytochemistry and Therapeutics (IMPPAT) database [14]. Initially, all the ligand molecules were submitted to ADMETlab 2.0 for ADMET-based screening. Out of 61 compounds, only 26 compounds were selected for molecular docking simulation, based on their ADMET properties. The ADMET profiling of all the compounds has been given in the Supplementary Materials (Supplementary Table S1). Saxagliptin was utilized as the control for the study, where its 3D structure was retrieved from the PubChem database. Protein and ligand structures were prepared for molecular docking simulation with AutoDock Tools 1.5.6 based on the previous study by the authors [15]. To prepare the protein structure, water and heteroatoms were removed. Polar hydrogens were added to stabilize the same. To minimize the proteins’ and ligands’ energy, Kollmann combined and Gasteiger charges were assigned. Before acquiring the ready structures for molecular docking simulation in PDBQT format, the AutoDock 4 atom type was assigned for each protein and ligand atom after energy minimization. The PDBQT format stores the protein’s ligands together with its atomic coordinates, partial charges, and AutoDock 4 atom type [15]. Literature analysis was used to predict the binding sites for the target protein [16]. The grid box for the protein’s binding pocket was set up using AutoDock Tools 1.5.6 measuring 17.106850 Å × 17.106850 Å × 17.106850 Å, coordinated at x = 15.677534 Å, y = 38.361920 Å, and z = 55.109795 Å, based on the position of the co-crystallized inhibitor ligand N7F901. The molecular docking simulation was validated according to a previous study, in which the authors used the same binding site (binding site of N7F901) [16].

Phytocompounds were virtually screened with the help of AutoDock Vina 1.1.2. It is the most sophisticated docking engine available in AutoDock Suite that is designed for receptor-ligand docking and operates on the Broyden–Fletcher–Goldfarb–Shannon (BFGS) algorithm to execute evaluation and ranking of envisaged ligand conformations exploiting scoring function [17,18]. Considering the high number of torsions occurring during the ligand preparation, a flexible-ligand docking approach was used for the molecular docking simulation, assuming the protein to be rigid while the ligand molecules are allowed 10 degrees of freedom. According to the binding affinity of generated poses, AutoDock Vina aligns them to compare displacement and conformational changes and reports results as root mean square deviation (RMSD) [19]. Therefore, out of ten, the first binding pose with zero RMSD and strongest binding affinity is deemed extremely genuine. Molecular docking simulation was visualized using Biovia Discovery Studio Visualizer 2021, an open-source visualization GUI tool. The extent of protein-ligand interaction was determined based on binding affinity, the total number of intermolecular interactions, and respective hydrophobic bonds [20,21]. In addition, re-docking of the 3 representative compounds (1S-α-pinene, β-pinene, and dehydro-*p*-cymene) from *O. tenuiflorum* was done using AutoDock 4.2., which uses the Lamarckian Genetic Algorithm (LGA) to execute evaluation and ranking of ligand conformations.

### 2.2. Molecular Dynamics Simulations

A command-line interface software package of GROMACS-2018.1 was used to perform molecular dynamics (MD) simulation. It is specifically designed for biochemical molecules such as proteins, lipids, and nucleic acids that possess a great deal of complex bonded interactions. For systems with hundreds to millions of particles, the program can simulate the Newtonian equations of motion, as well as calculate nonbonded interactions swiftly. Based on a previous study, docked complexes of DPP4 protein with saxagliptin, 1S-α-pinene, β-pinene, and dehydro-*p*-cymene having the most negative binding affinities were submitted for simulation [22]. The CHARMM36 force field was used to approximate the ligand structures, and the SwissParam server was used to generate the ligand topology. On the other hand, protein structure was also added with the CHARMM36 forcefield using the pdb2gmx module. Further, a 5000-step energy minimization in vacuum using the steepest descent algorithm was done. The distance between each protein complex and the box’s edges was kept at 10 Å. In order to maintain the necessary 0.15 M salt concentration, the solvent was incorporated into the TIP3P water model along with the appropriate number of Na^+^ and Cl^−^ counterions. The simulation boxes consisting of protein–ligand complexes were simulated for 100 ns at 310 K temperature and 1 bar pressure. In addition, apoprotein structure was also submitted for the simulation in the same environment [22]. In total, 5 simulations were performed (4 protein–ligand complexes and 1 apoprotein). We performed a trajectory analysis of root-mean-square deviation (RMSD), root-mean-square-fluctuation (RMSF), radius of gyration (Rg), ligand-hydrogen bonds, and solvent accessible-surface-area (SASA) parameters and plotted the results using QtGRACE, a GUI based software for plotting the results of MD simulation [23].

### 2.3. Binding Free Energy Calculations

Using the MD simulation results, all the protein–ligand complexes were subjected to binding free energy calculations using the Molecular Mechanics/Poisson-Boltzmann Surface Area (MM-PBSA) technique. It is an efficient and reliable free energy simulation method to model molecular recognition, such as for protein-ligand binding interactions. A GROMACS programme, g_mmpbsa with the MmPbSaStat.py script was exploited to evaluate the binding free energy for each protein–ligand complex [24]. g_mmpbsa calculates binding free energy using three components: molecular mechanical energy, polar and apolar solvation energies, and molecular mechanical energy. The binding free energy was computed using the molecular dynamics trajectories of the last 50 ns and dt 1000 frames. The Equations (1) and (2) are used to calculate the free binding energy [25].

ΔG_Binding_ = G_Complex_ − (G_Protein_ + G_Ligand_) (1)

ΔG = ΔE_MM_ + ΔG_Solvation_ − TΔS = ΔE_(Bonded+non-bonded)_ + ΔG_(Polar+non-polar)_ − TΔS (2)

G_Binding_: binding free energy; G_Complex_: total free energy of the protein–ligand complex; G_Protein_ and G_Ligand_: total free energies of the isolated protein and ligand in solvent, respectively; ΔG: standard free energy; ΔE_MM_: average molecular mechanics potential energy in vacuum; G_Solvation_: solvation energy, ΔE: total energy of bonded as well as non-bonded interactions; ΔS: change in entropy of the system upon ligand binding; T: temperature in Kelvin [26,27].

### 2.4. Druglikeliness and Pharmacokinetics Analyses

In order to conduct pharmacokinetic analyses, chemical structures of 1S-α-pinene, β-pinene, dehydro-*p*-cymene, and saxagliptin were submitted to the ADMETlab 2.0 server in SMILES format. ADMETlab 2.0 is an integrated online platform for accurate, comprehensive and systematic evaluation of ADMET properties. Parameters such as drug half-life (HL < 3 h), Lipinski’s rule (LR) of five, MDCK cell permeability, Caco-2 permeability, volume distribution, plasma protein binding, Cytochrome P inhibition, clearance, hERG inhibition, human hepatotoxicity, and carcinogenicity were used to appraise the pharmacokinetics of ligand molecules [28].

## 3. Results

### 3.1. Molecular Docking Simulations

**Figure 1:**
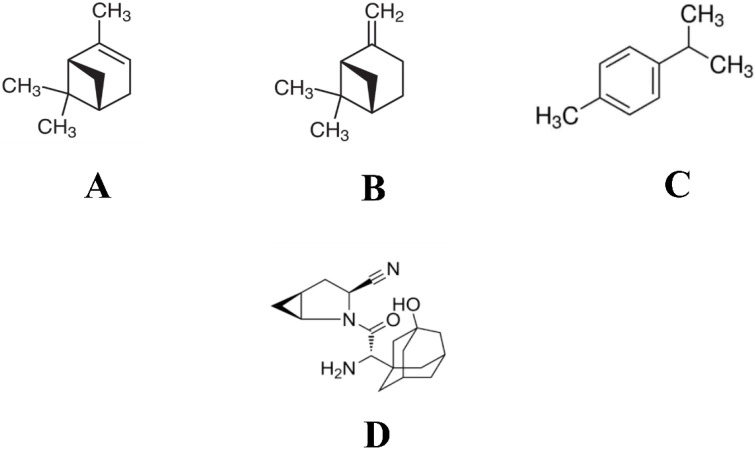
2D structures of representative compounds selected from the virtual screening of *O. tenuiflorum* phytoconstituents. (**A**) 1S-α-pinene, (**B**) β-pinene, (**C**) dehydro-*p*-cymene, and (**D**) saxagliptin.

Among all the 26 phytocompounds docked with DPP4 protein, 1S-α-pinene, β-pinene, and dehydro-*p*-cymene were selected for further analysis, as the three compounds were predicted with higher binding efficiency. All the compounds were reported to bind with the inhibitor binding site of the DPP4 protein, within the binding site of the co-crystallized inhibitor compound N7F901. The 2D structures of these representative compounds have been given in Figure 1. The criteria set for selecting the most potential inhibitors was based on the binding affinity, total number of intermolecular interactions, and total number of hydrophobic interactions. Virtual screening of *O. tenuiflorum* phytoconstituents and saxagliptin against DPP4 is represented in Table 1.

1S-α-pinene was found to interact with the residues from α/β hydrolase domain. In total, nine intermolecular interactions, with all of them being hydrophobic. The compound bound with Tyr666 (3.89 Å) through a π-sigma bond. It also formed an alkyl bond with Val656 (4.56 Å). Further, there were seven π-alkyl bonds with Tyr631 (5.08 Å), Trp659 (4.82 Å), Tyr662 (4.72 Å), Tyr662 (4.23 Å), Tyr666 (5.02 Å), Tyr666 (5.41 Å), and Tyr666 (4.28 Å). With these intermolecular interactions, 1S-α-pinene was bound with an affinity of −6.2 kcal/mol. Further, β-pinene made eight intermolecular interactions with the binding residues of α/β hydrolase domain. It formed a π-sigma bond with Tyr666 (3.73 Å) and an alkyl bond with Val656 (4.69 Å). It formed six π-alkyl bonds with Tyr631 (5.20 Å), Trp659 (4.89 Å), Tyr662 (4.70 Å), Tyr666 (5.05 Å), Tyr666 (4.14 Å). With these interactions, β-pinene was able to bind with an affinity of −6.2 kcal/mol.

Furthermore, dehydro-*p*-cymene was able to form 11 hydrophobic intermolecular interactions. It formed a single π-π stacked interaction with Tyr666 (4.07 Å), three alkyl bonds with Val656 (5.05 Å), Val711 (4.68 Å), and Val656 (4.49 Å). In addition, it formed seven π-alkyl bonds with Phe357 (5.16 Å), Tyr631 (5.21 Å), Trp659 (4.69 Å), Tyr662 (4.39 Å), Tyr662 (4.03 Å), Tyr666 (5.34 Å), and Tyr666 (4.20 Å). In parallel, the binding affinity of saxagliptin was found to be −7.4 kcal/mol. It formed four π-alkyl bonds with Tyr547 (5.28 Å), Tyr662 (5.24 Å) and Tyr666 (4.71 Å and 5.34 Å) as well as two hydrogen bonds with Tyr547 (1.94 Å) and Glu206 (2.19 Å).

**Figure 2:**
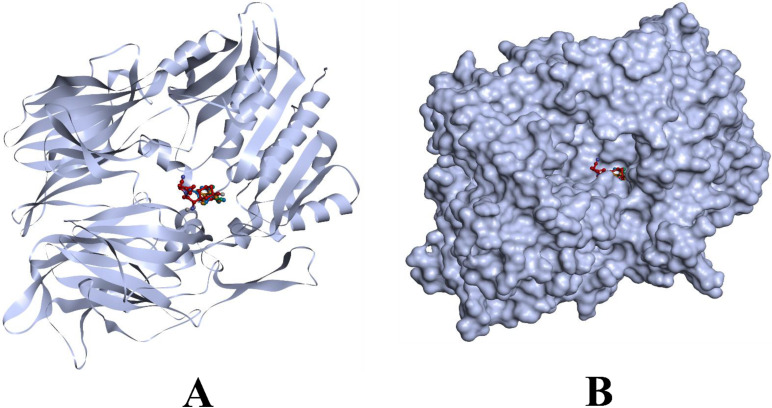
Visualization of binding of representative compounds of *O. tenuiflorum* and saxagliptin with DPP4 inside the inhibitor binding pocket of the protein. (**A**) Bound chemicals inside the protein binding pocket (ribbon representation), (**B**) bound chemicals inside the protein binding pocket (surface representation). Orange: 1S-α-pinene, green: β-pinene, blue: dehydro-*p*-cymene, and red: saxagliptin.

**Figure 3:**
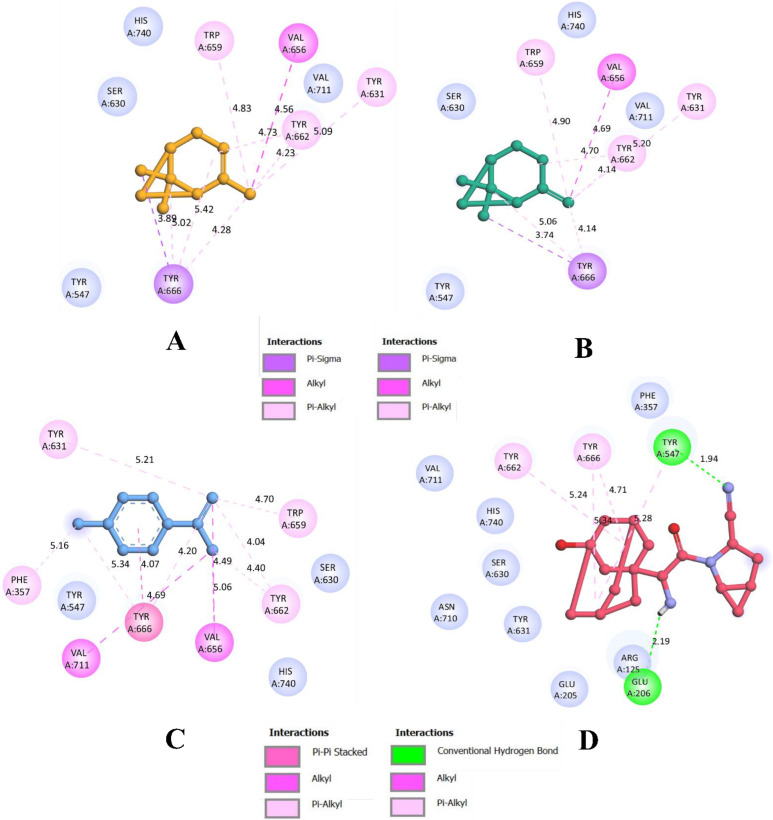
Visualization of binding interaction of representative compounds of *O. tenuiflorum* and saxagliptin with DPP4 in 2D; (**A**) binding interactions of 1S-α-pinene, (**B**) binding interactions of β-pinene, (**C**) binding interactions of dehydro-*p*-cymene, and (**D**) binding interactions of saxagliptin. Colored: bound residues, blue: surrounding residues.

Results from molecular docking simulation reveal that all the compounds bound with the residues from the binding pocket. Since they bind within the inhibitor binding site previously occupied by the co-crystallized inhibitor compound N7F901, they are predicted to be the potential inhibitors of the DPP4 proteins. Even though all the ligands occupied the same binding site, dehydro-*p*-cymene had a better interaction than saxagliptin due to the higher number of hydrophobic bonds. Figure 2 and Figure 3 visualize the interaction of selected phytocompounds and saxagliptin with DPP4 in 3D and 2D, respectively. In addition, results from re-docking and rescoring of the representative compounds from *O. tenuiflorum* have been given in the Supplementary Materials (Supplementary Figure S1). These results indicate that re-docked conformations are in accordance with the previous docking results obtained using AutoDock Vina 1.1.2.

### 3.2. Molecular Dynamics Simulation

The docking analysis is validated using a molecular dynamics simulation, which also reveals the stability of the docked complex and the target protein. As a result, it becomes crucial to carry out molecular dynamics simulation following docking simulation. MD simulation determines the stability, conformational flexibility, and dynamics of drug-target complexes at the atomic and molecular level, through the analysis of a variety of trajectories.

We investigated several parameters, such as the protein–ligand complex RMSD, RMSF, Rg, SASA, and ligand-hydrogen bonds to evaluate the complex’s overall stability. The root-mean-square deviation (RMSD) graph represents the stability of ligands within the binding pocket of the receptor during a simulation of 100 ns. On the contrary, root-mean-square fluctuation (RMSF) provides a measure of the average deviation of a particle (e.g., a protein residue) over time from a reference position that identifies which portions of the structure are fluctuating most or least from their mean. RMSF values directly influence the binding poses and interactions. The Radius of Gyration (Rg) provides information on the compactness and size of the protein molecules throughout the simulation. The measurement of the distance between the center of mass of the protein and both of its termini gives an understanding of how regular secondary structures are incorporated into protein 3D structures. In addition, the changes in the accessibility of protein to solvent can be determined by computing the solvent accessible surface area (SASA). It measures the area surrounding a protein–ligand complex’s hydrophobic core. A rapid and accurate calculation of SASA is extremely useful in the analysis of biomolecules. Furthermore, in reference with the 100 ns simulation, we calculated and plotted a varying number of ligand-hydrogen bonds since it plays a crucial role in dynamic trajectory analysis and is necessary for determining the structural re-agreement.

In case of dehydro-*p*-cymene complexed with the DPP4 protein, the molecule stayed in the inhibitor binding site till the end of the simulation run. The RMSD plot showed that the molecule gained stability after 30 ns, and became concurrent with the RMSD plot of apoprotein after 50 ns. The compound was not found with any of the unusual fluctuations in case of RMSF analysis. This shows that the compound was stable throughout the simulation run. The fluctuation pattern of apoprotein and protein-dehydro-*p*-cymene was similar. Except for the loop region (200–300 residues), only minimal fluctuations were observed throughout the simulation, including the N- and C-terminals of the protein–ligand complex molecule. In addition, the Rg plot shows that dehydro-*p*-cymene was compactly bound to the protein. This binding resulted in a significant decrease of the SASA value of the protein–dehydro-*p*-cymene complex. Since the compound efficiently occupies the inhibitor binding site, the available SASA decreases upon the extension of the simulation period. Further, analysis of ligand hydrogen bond shows that 1S-α-pinene forms a maximum number of hydrogen bonds than all the experimental compounds, including saxagliptin. The pattern of results obtained for other representative compounds of *O. tenuiflorum* (1S-α-pinene and β-pinene) was in accordance with that of 1S-α-pinene. Both β-pinene and dehydro-*p*-cymene were stable inside the inhibitor binding site of the DPP4 protein.

**Figure 4:**
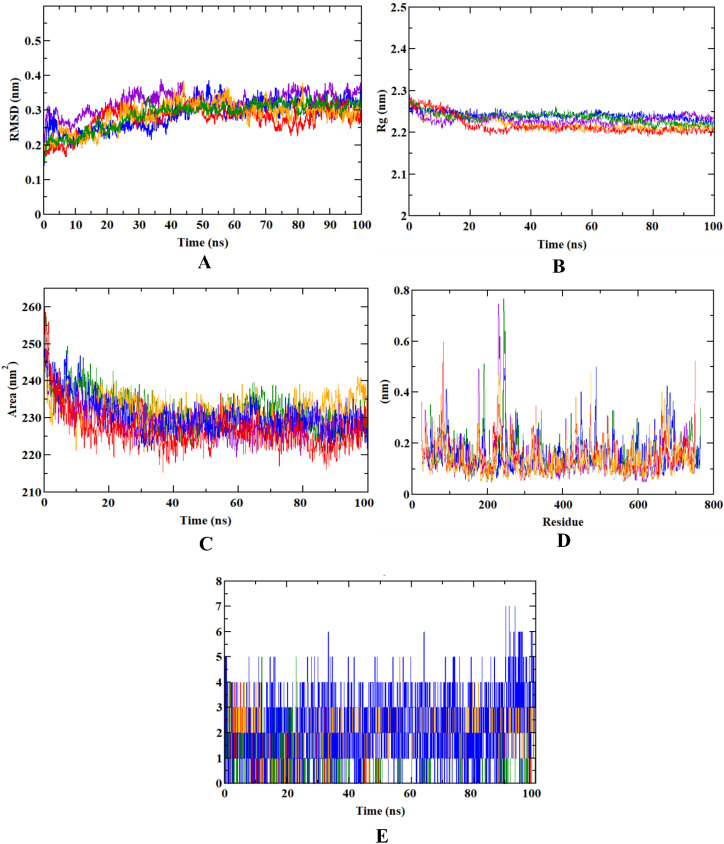
Visualization of MD trajectories of experimental compounds complexed with DPP4 protein obtained from the MD run of 100 ns. (**A**) protein–ligand complex RMSD, (**B**) protein–ligand complex Rg, (**C**) protein–ligand complex SASA, (**D**) protein–ligand complex RMSF, (**E**) ligand-hydrogen bonds. (Violet: apoprotein; orange: protein–1S-α-pinene complex; green: protein–β-pinene complex; blue: protein–dehydro-*p*-cymene complex; red: protein–saxagliptin complex).

However, all the plots obtained from the trajectories of saxagliptin showed that the compound was comparatively unstable. Although it occupies the same inhibitor binding site, similar to the other compounds, values of trajectory analysis show that the compound is unstable in comparison with other compounds. Among the three representative compounds from *O. tenuiflorum*, dehydro-*p*-cymene was more stable throughout the simulation, although the other two compounds (1S-α-pinene and β-pinene) were nearly as stable as the previous one. Table 2 depicts the average MD trajectory values (RMSD, RMSF, Rg, and SASA) obtained for DPP4 apoprotein as well as DPP4 protein complexed with different experimental molecules, whereas Figure 4 visualizes the trajectory plots obtained from the simulation run.

### 3.3. Binding Free Energy Calculations

Binding free energy calculations offer an attractive approach to predict the protein–ligand binding affinities. Using molecular simulations and statistical mechanics, it calculates the energy released during the formation of the complex. In this scenario, several parameters such as Van der Waals, electrostatic, polar solvation, SASA, and binding energy were implemented in the calculation of binding free energy. Van der Waal’s energy is primarily used by both ligands for protein complexation. In terms of stability, the protein–dehydro-*p*-cymene complex had an edge over the other protein–ligand complexes as well as protein–saxagliptin complex. Though all the complexes majorly used Van der Waal’s energy to form, binding energy also significantly contributed to the formation of these complexes.

The Van der Waal’s energy for the protein–dehydro-*p*-cymene (−52.918 ± 5.670 kJ/mol) was found to be more negative than that of protein–saxagliptin complex (−39.920 ± 24.183 kJ/mol). In all the types of binding free energies, the protein–dehydro-*p*-cymene complex was found to have higher efficiency. Other representative compounds (1S-α-pinene and β-pinene) had similar patterns of binding efficiency, yet higher than saxagliptin. Furthermore, standard deviations of polar solvation, SASA, and binding energies demonstrate the higher deviation of saxagliptin from the expected value. This indicates that saxagliptin is less stable than other protein–ligand complexes during its formation. Results from binding free energy calculations reveal that all the *O. tenuiflorum* phytoconstituents are comparatively stable during molecular dynamics simulation runs than saxagliptin. These results also support the outcomes from molecular docking and dynamics simulation. Table 3 summarizes the binding free energy calculation done for protein–ligand complexes.

### 3.4. Druglikeliness and Pharmacokinetics Analysis

In silico pharmacokinetics analysis helps to understand how a molecule behaves once in the body. The study of pharmacokinetics involves the dynamic movements of xenobiotics inside the body including the kinetics of absorption, distribution, biotransformation/metabolism excretion and (ADMET). The druglikeness of the compounds was assessed using Lipinski’s rule of five, which includes parameters like molecular weight, topological polar surface area (TPSA), hydrogen bond donors, hydrogen bond acceptors, Nb rotatable bonds. All the compounds subjected for pharmacokinetics including saxagliptin excel on Lipinski’s rule of five.

**Figure 5:**
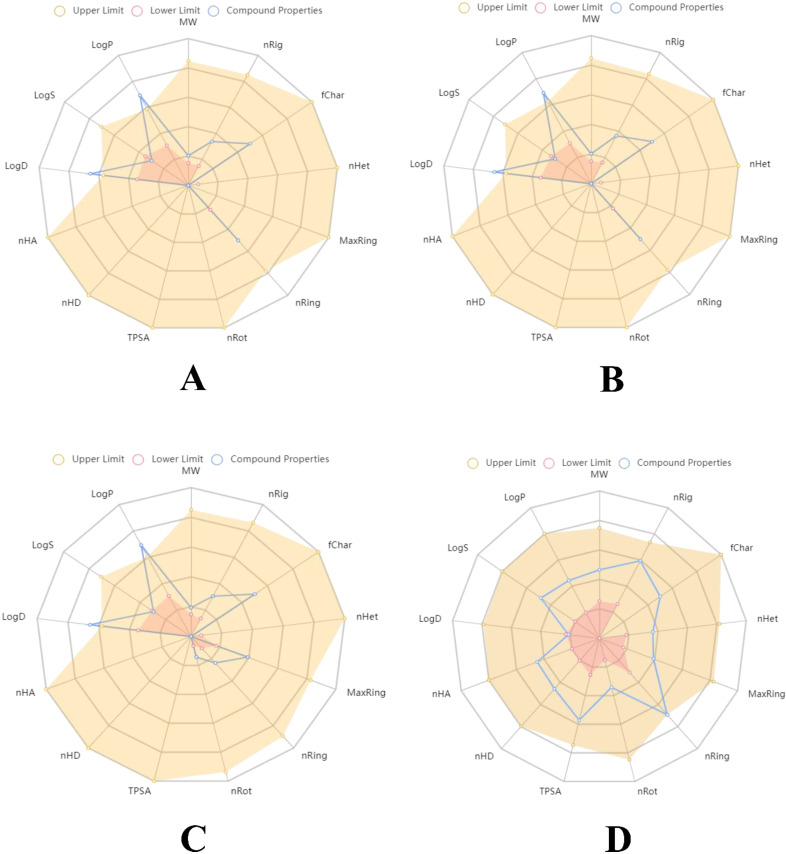
Pharmacokinetic mapping of representative compounds of *O. tenuiflorum* and saxagliptin; (**A**) 1S-α-pinene, (**B**) β-pinene, (**C**) dehydro-*p*-cymene, and (**D**) saxagliptin (obtained from ADMETlab 2.0).

In terms of ADMET properties, all the compounds accomplished good results for MDCK Permeability, volume distribution, the fraction unbound in plasma (Fu), CYP inhibition, clearance and hERG inhibition. 1S-α-pinene had higher distribution compared to other compounds. The clearance parameter also showed a similar pattern, where 1S-α-pinene had an edge over the other compounds. Although saxagliptin showed comparatively higher values in adsorption and metabolism parameters, the compound showed negative results for Caco-2 Permeability and other toxicity tests including human hepatotoxicity and carcinogenicity. As a result, saxagliptin is predicted to have toxic and carcinogenic properties, causing damage to the liver and respiratory system. However, selected phytoconstituents from *O. tenuiflorum* are not predicted to have any carcinogenic or toxic properties. These results show that *O. tenuiflorum* phytocompounds have drug-like properties, which could be evaluated using animal models and clinical trials. Druglikeness and pharmacokinetics studies are tabulated in Table 4 illustrate the pharmacokinetic outcomes of selected *O. tenuiflorum* phytoconstituents and saxagliptin. The pharmacokinetic mapping of these compounds has been described in Figure 5.

## 4. Discussion

Even after decades, T2DM remains a major concern in healthcare worldwide. The glucose-lowering therapy, in conjunction with a healthy lifestyle, is a mainstay of glycemic control and prevention of diabetes-related complications. However, numerous insulin preparations and several synthetic oral antidiabetic drugs have not eliminated the need for discovery and development of novel antidiabetic drugs. The development of resistance and adverse effects associated with long-term use of these drugs has led to an active area of research for improving the glucose-lowering efficacy of future diabetes medications [29,30].

Contrary to conventional synthetic drugs, phytocompounds are evolving as new leads for the development of novel drugs. Phytocompounds exhibit a wide range of biological properties and show the least unwanted side effects [31,32,33]. Taking this into account, we investigated phytocompounds from *Tulsi* (*O. tenuiflorum*) to study their potential as antidiabetic drugs. The culinary, medicinal, and industrial importance of this plant prompted researchers to explore its chemical and pharmacological properties. In vitro antidiabetic and antihyperlipidemic effects of *O. tenuiflorum* phytoconstituents have already been explored [34]. Using the fixed oil obtained from fresh *O. tenuiflorum* leaves, it was observed to significantly lower diabetically elevated blood glucose levels, serum lipid profiles, and serum insulin levels in streptozotocin-induced type 1 diabetes mellitus rats within three weeks [35]. Further, a study revealed that *Tulsi* is safe for humans, suggesting its safe herbal intervention that may assist in normalizing glucose, blood pressure, and lipid profiles, and dealing with psychological and immunological stress [36]. Thus, sufficient evidence motivated us to explore the antidiabetic potential of *Tulsi* phytocompounds in the treatment of diabetes mellitus. However, phytochemical profiling and discovery of specific compounds that are responsible for antidiabetic pharmacological action is yet to be deciphered. Since in silico techniques reveal potential compounds with specific antidiabetic pharmacological action at the molecular level, it is essential to screen phytocompounds against the target proteins before going for in vitro and in vivo studies. Also, the action of these phytochemicals on DPP4 is not yet evaluated.

This study considers DPP4 as a promising therapeutic target for diabetes mellitus. DPP4 inhibitors provide better glycemic control over longer periods of time than early oral hypoglycemics. The most widely used DPP4 inhibitors include sitagliptin, linagliptin, saxagliptin, alogliptin, vildagliptin, anagliptin, gemigliptin, and teneligliptin; these are implemented into the treatment algorithms of T2DM in many national and international guidelines [8,37]. However, the pharmacokinetic considerations and adverse effects of these synthetic DPP4 inhibitors remain a major concern. The most common catastrophic effects of current DPP4 inhibitors are upper respiratory tract infection, nasopharyngitis, headache and urinary tract infection. At present, no phytocompound-based DPP4 inhibitors are commercially available to replace these inimical inhibitors. Our study impetus was to earmark three phytoconstituents from *O. tenuiflorum* as potential DPP4 inhibitors in the treatment of T2DM (1S-α-pinene, β-pinene, and dehydro-*p*-cymene). We used saxagliptin as a control to compare the efficiency of the *O. tenuiflorum* representative compounds.

The whole phytocompound library of *O. tenuiflorum,* containing 26 chemical structures, was docked with the targeted DPP4 protein of humans. From the structure-based virtual screening of the compounds, 1S-α-Pinene, β-Pinene, and dehydro-*p*-cymene were selected as lead compounds for the further computational applications due to their overall excellence in terms of binding affinity, the total number of intermolecular interactions, and the total number of hydrophobic bonds. During the molecular docking simulation, 1S-α-pinene, β-pinene, and dehydro-*p*-cymene exactly occupied the inhibitor binding pocket of the targeted protein located between the lipophilic 8-bladed β-propeller domain and hydrophobic α/β hydrolase domain. They successfully bound to the inhibitor binding site previously occupied by the co-crystallized inhibitor ligand N7F901. The binding interactions formed by the *O. tenuiflorum* compounds are similar to those observed in a previous study [16]. However, saxagliptin was also bound to the same binding site, yet with lower binding efficiency compared to the *O. tenuiflorum* phytoconstituents. The binding of *O. tenuiflorum* compounds to the inhibitor binding site of the protein also resembled the docking results obtained in a previous study [38], where synthesized Schiff’s based compounds were docked into the inhibitor binding site of the DPP4 protein structure. The compounds were also evaluated in vitro, and were found with favorable results. Since the binding interactions of these Schiff’s based compounds and *O. tenuiflorum* compounds selected from our study are similar, the latter could act as the potential inhibitors of the enzyme. Therefore, employing 1S-α-pinene, β-pinene, and dehydro-*p*-cymene could bring up the essential biological inhibition of DPP4 [39,40]. In a similar approach, three phytochemicals isolated from the seeds of *Lens culinaris* screened against DPP4, but the study did not include molecular dynamics simulation and binding free energy calculations to validate their lead compounds [41]. Since these studies have not reported the stability of the reported potential inhibitor compounds, the validation of the compounds remains undone. Therefore, in our study, we have employed the molecular dynamics simulation technique to elucidate the same.

During the 100 ns long molecular dynamics simulation, all the trajectories of the representative *O. tenuiflorum* compounds complexed with the DPP4 protein were found to be stable throughout the simulation. However, dehydro-*p*-cymene was comparatively more stable than all the reported compounds. All the parameters—RMSD, RMSF, SASA, Rg, and ligand hydrogen bonds—suggest the higher stability, compactness, extensive interaction and minimal fluctuation of dehydro-*p*-cymene with the target protein; in comparison to the saxagliptin, dehydro-*p*-cymene remained stable in the inhibitor binding pocket during the simulation process. The simulation data shows that both dehydro-*p*-cymene and saxagliptin penetrated the active site and perform stable interactions, probably contributing to their biological activity. However, dehydro-*p*-cymene was found to be comparatively better than saxagliptin in all the parameters considered for simulation studies. A comparative account of binding interactions during molecular docking and dynamics simulation studies conducted for experimental compounds was also done (Supplementary Table S2), where Tyr666 was found to be the common binding residue for all the experimental molecules (Supplementary Table S3). Therefore, Tyr666 could be used as a key binding residue in future studies. Comparing the molecular dynamics simulation outcomes with a previous study, we found the dehydro-*p*-cymene exhibits better stability, less fluctuation and a higher degree of concordance and compactness than Coagulin L, a reported potential inhibitor of DPP4 [42]. Further, the binding free energy calculations revealed all the protein–ligand complexes were formed majorly using Van der Waal’s energy. However, binding energy also predicted with higher contribution in the complex formation. Standard deviations calculations indicate that the obtained values for different categories of binding free energy calculations are within the expected range or very near to the normal value. The cumulative findings of structure-based virtual screening, molecular dynamics simulation and MM-PBSA approaches validate the efficacious nature of our lead, dehydro-*p*-cymene. A study reported three potential phytocompounds from the library of *Moringa oleifera* based on in silico pharmacokinetic analysis, which was the prime analysis to select the representative compounds [43]. However, the study did not demonstrate the stability and dynamics of the ligand–receptor complex through molecular dynamics simulation and binding free energy calculations.

Furthermore, in terms of pharmacokinetic analysis, dehydro-*p*-cymene and representative *O. tenuiflorum* compounds outperformed saxagliptin. Saxagliptin is predicted with potential to cause liver damage, respiratory toxicity and as the menace of cancer. The smaller size and low molecular weight of the *O. tenuiflorum* compounds help them to be readily absorbed by the body and provide an appropriate volume of distribution. Nevertheless, the higher molecular weight (315.19 g/mol) of saxagliptin restricts its Caco-2 permeability and predicts low intestinal absorption and distribution. Also, saxagliptin has been reported with several adverse effects in clinical investigations on diabetic individuals. The drug is chiefly associated with the adverse effects on cardiovascular health [44]. Its prolonged usage in diabetic individuals has resulted in upper respiratory tract infection, nasopharyngitis, nonfatal myocardial infarction and sometimes cardiovascular death. It is also linked with nonfatal stroke, urinary tract infection, development of severe headache, and low risk of hypoglycemia [44,45]. A clinical trial conducted on diabetic individuals using saxagliptin reported the incidence of pancreatitis and pancreatic cancer in few patients [46]. These outcomes support the pharmacokinetic results obtained in this study. In addition, it is difficult for the high molecular weight molecules such as Coagulin L (650.8 g/mol) to be absorbed and distributed in the body. As the molecular weight increases, the absorption decreases [47,48]. In contrast, due to their low molecular weight, *O. tenuiflorum* compounds were able to meet the pharmacokinetic parameters of absorption, distribution, metabolism, excretion, and toxicity.

Recent studies investigated the antidiabetic action of plants like *Foeniculum vulgare*, *Eryngium carlinae*, and *Pistacia atlantica*, where both 1S-α-pinene and β-pinene have been proved with antioxidant and anti-hyperglycemic potential in diabetic rats [49]. These monoterpenoids have also been reported with gastroprotective, anxiolytic, and antitumor effects [50]. Although both of these compounds have been linked with anti-diabetic effects, they have been used against carbohydrate digestive enzymes like α-glucosidase and α-amylase [51]. Also, the 3rd representative compound from *O. tenuiflorum*, dehydro-*p*-cymene, is yet to be thoroughly investigated for its antidiabetic potential using in vitro and in vivo models. Our study provides a deep insight into the interaction of *O. tenuiflorum* compounds with DPP4 and validates their novelty as the potential inhibitors of DPP4, targeting antidiabetic effects for the first time. Also, our phytoinformatics-based research compares favorably with all the above cited studies based on its novelty, design, experimentation, and outcomes. In this context, we can conclude that our study has identified *O. tenuiflorum* compounds (1S-α-pinene, β-pinene, and dehydro-*p*-cymene) as the potential novel inhibitors for DPP4, highlighting the pharmacological significance of *O. tenuiflorum* against diabetes mellitus.

## 5. Conclusions

Due to the absence of information about plant-based medicinal therapeutics, the pharmaceutical industries lack interest in phytochemical research compared with synthetic drugs inhibiting DPP4. In this context, we investigated the antidiabetic potential of *O. tenuiflorum* phytocompounds against DPP4 through the in-silico approach. In addition to molecular docking, molecular dynamics simulations, and binding free energy calculations, we analyzed druglikeness and pharmacokinetics of the experimental molecules. In the virtual screening, 1S-α-pinene, β-pinene, and dehydro-*p*-cymene were found to be the potential inhibitors of DPP4 with the highest binding affinity and interaction for the target protein. Additionally, molecular dynamics simulations and binding free energy calculations indicate greater stability of these compounds than saxagliptin. Pharmacokinetic studies also support these representative compounds of *O. tenuiflorum* as non-toxic and non-cancerous. On the contrary, saxagliptin was found to be comparatively unstable during molecular dynamics studies. Pharmacokinetic analysis of saxagliptin also reveals that saxagliptin is toxic and cancerous. Results from our pharmacokinetic studies were found to be in accordance with the clinical trials of saxagliptin, where the drug was found to cause pancreatitis and pancreatic cancer. However, few in vitro and in vivo studies reported the antidiabetic potential of 1S-α-pinene and β-pinene. Further, the antidiabetic properties of dehydro-*p*-cymene are yet to be elucidated. From these outcomes, we conclude that 1S-α-pinene, β-pinene, and dehydro-*p*-cymene could act as a potential inhibitor of the DPP4, and dehydro-*p*-cymene as the lead inhibitor. The reported compounds could be evaluated through in vitro and in vivo approaches to discover novel phytochemical-based inhibitors of DPP4.

## Figures and Tables

**Table 1:** Virtual screening of *O. tenuiflorum* phytocompounds and saxagliptin against DPP4.

Sl. No.	Name of the Compound	Binding Affinity (kcal/mol)	Total No. of Intermolecular Interactions	Total No. of Hydrophobic Bonds
1	(−)-Alloaromadendrene	−6.3	4	4
2	(−)-Camphene	−5.4	2	2
3	(−)-Linalool	−5.7	11	10
4	(+)-α-Phellandrene	−5.9	6	6
5	(+)-Endo-β-bergamotene	−6.5	6	6
6	(1S)-1,7,7-Trimethylbicyclo[2.2.1]heptan-2-one	−5.7	2	2
7	(1S, 2R, 4S)-(−)-Bornyl acetate	−6.2	1	0
8	(E)-β-ocimene	−5.4	9	9
9	1S-α-pinene	−6.2	9	9
10	2,3-Dimethylaniline	−5.5	2	2
11	3-Carene	−6.1	6	6
12	4-Terpineol	−6.1	3	3
13	Acetyleugenol	−6.2	6	6
14	α-Fenchene	−5.8	7	7
15	α-Terpineol	−5.8	8	7
16	β-Caryophyllene	−6.0	2	2
17	β-Pinene	−6.2	8	8
18	*Cis*-Anethole	−5.8	6	6
19	Cyclo-(L-Val-L-Leu)	−6.6	6	6
20	Dehydro-*p*-cymene	−6.4	11	11
21	Eucalyptol	−6.1	1	1
22	γ-Selinene	−6.6	3	3
23	Geranyl Acetate	−6.1	5	5
24	Isoeugenol	−5.6	7	7
25	Myrcene	−5.5	10	10
26	Phytosterols	−8.3	3	2
Control	Saxagliptin	−7.4	6	4

**Table 2:** MD trajectory values obtained for experimental compounds complexed with DPP4 protein during MD simulation.

MD Trajectory Plots	RMSD (nm)	RMSF (nm)	Rg (nm)	SASA (nm^2^)	Number of Ligand H-Bonds
Apoprotein	0.321	0.262	2.341	236.25	
DPP4-1S-α-pinene complex	0.287	0.291	2.349	237.25	5
DPP4-β-pinene complex	0.285	0.245	2.296	239.60	5
DPP4-dehydro-*p*-cymene	0.291	0.259	2.340	235.10	7
DPP4-saxagliptin complex	0.232	0.250	2.310	231.12	4

Note: Apoprotein is only a protein moleculae simulated without any ligand complexed with it. Therefore, there will be no hydrogen bonds formed by the ligand with the protein during the molecular dynamics simulation.

**Table 3:** Binding free energy calculations of representative compounds of *O. tenuiflorum* and saxagliptin complexed with DPP4 protein.

Types of Binding Free Energies	DPP4-1S-α-Pinene Complex (kJ/mol)	DPP4-β-Pinene Complex (kJ/mol)	DPP4-Dehydro-*p*-Cymene (kJ/mol)	DPP4-Saxagliptin Complex (kJ/mol)
Van der Waal energy	−51.461 ± 10.125	−49.871 ± 9.012	−52.918 ± 5.670	−39.920 ± 24.183
Electrostatic energy	−1.028 ± 9.716	−0.389 ± 12.133	−1.209 ± 6.999	−0.214 ± 1.305
Polar solvation energy	32.514 ± 12.871	28.574 ± 20.445	21.841 ± 10.001	22.912 ± 35.181
SASA energy	−3.898 ± 2.999	−2.901 ± 2.101	−4.996 ± 7.120	−4.614 ± 3.652
Binding energy	−34.807 ± 11.099	−27.569 ± 13.122	−33.124 ± 14.909	−29.752 ± 48.193

**Table 4:** Druglikeness and pharmacokinetic evaluation of representative compounds of *O. tenuiflorum* and saxagliptin.

Categories	Types of Parameters	1S-α-Pinene	β-Pinene	Dehydro-*p*-Cymene
Druglikeliness based on Lipinski’s rule of five	Molecular weight	136.13 g/mol	136.13 g/mol	315.19 g/mol
	Topological polar surface area (Å)	0.0	0.0	90.35
	No. of hydrogen bond donors	0	0	3
	No. of hydrogen bond acceptors	0	0	5
	No. of rotatable bonds	0	0	3
Adsorption	Caco-2 Permeability	−4.303	−4.46	−5.212
	MDCK Permeability	1.8e − 05	2e − 05	9.4e − 05
Distribution	Volume distribution	1.73	1.091	1.426
	Plasma Protein Binding	86.33%	64.33%	8.648%
Metabolism	CYP1A2 inhibition	0.469	0.296	0.006
	CYP2C19 inhibition	0.267	0.163	0.029
	CYP2C9 inhibition	0.312	0.321	0.039
	CYP2D6 inhibition	0.012	0.009	0.019
	CYP3A4 inhibition	0.034	0.026	0.338
Excretion	Clearance	15.022	10.097	8.981
	Half-life (T 1/2)	0.114	0.107	0.187
Toxicity	Human Hepatotoxicity	0.196	0.109	0.912
	hERG inhibition	0.006	0.005	0.013
	Carcinogenicity	0.056	0.042	0.919

Note: Molecular weight (<500: good); Topological polar surface area (<150: good); Number of rotatable bonds; Number of hydrogen bond acceptors (≤10: good); Number of hydrogen bond donors (≤5: good); MDCK cell permeability (low: <2 × 10^−6^ cm/s, medium: 2–20 × 10^−6^ cm/s, high: >20 × 10^−6^ cm/s); Caco-2 Permeability (Optimal: higher than −5.15 log unit); Volume distribution (Optimal: 0.04–20 L/kg); Protein-plasma binding (Optimal: <90%); Cytochrome P inhibition (>0.0 or positive value: good); Clearance (High: >15 mL/min/kg; moderate: 5–15 mL/min/kg; low: <5 mL/min/kg); drug half-life (Category 1: long half-life; Category 0: short half-life; long half-life: >3 h; short half-life: <3 h); hERG inhibition (0.0 or negative value: good); AMES toxicity (0.0 or negative value: good); Human Hepatotoxicity; Carcinogen city; Respiratory Toxicity (Category 1: toxicants; Category 0: non-toxicants).

## Data Availability

Not applicable.

## Supplementary Materials

The following supporting information can be downloaded at: https://www.mdpi.com/article/10.3390/molecules27165133/s1. Table S1: ADMET profiling of *O. tenuiflorum* phytocompounds; Figure S1: Binding interactions of A) 1S-α-pinene (binding affinity: −5.3 kcal/mol), B) β-pinene (binding affinity: −5.2 kcal/mol), and C) Dehydro-*p*-cymene (binding affinity: −5.5 kcal/mol) with DPP4 protein; Table S2: Comparative account of binding interactions during molecular docking and dynamics simulation studies conducted for experimental compounds; Table S3: Common binding interactions observed between molecular docking and dynamics simulation studies conducted for experimental compounds.

## Sample Availability

Samples of the compounds are not available from the authors.
