# Supplementary material for: Computer-Aided Screening of Phytoconstituents from Ocimum tenuiflorum against Diabetes Mellitus Targeting DPP4 Inhibition: A Combination of Molecular Docking, Molecular Dynamics, and Pharmacokinetics Approaches
Source: Molecules. 2022 Aug 12;27(16):5133. doi: 10.3390/molecules27165133 (PMC9415412; doi:10.3390/molecules27165133)
Supplement: Supplementary file 1 [file molecules-27-05133-s001.zip › molecules-1785676-supplementary.pdf]

Supplementary Material

Supplementary Table S1: ADMET profiling of *O. tenuiflorum* phytocompounds

Sl. No	Compound Names	Oral bioavailability (OB≥30%)	Blood-brain barrier (BBB)	Drug half-life (HL<3h)	Lipinski's rule (LR) of five	Intestinal epithelial permeability (Caco-2 cells)	Drug-induced liver injury (DILI)	Clearness (CL>15 ml/min/kg)	Molecular Weight (MW 100~600)	Hydrogen bond acceptor (0~12)	Hydrogen bond donor (0~7)	TPSA (0~140)	PAINS
1	Cyclo (L-val-L-Leu)	Pass	Pass	0.671	Accepted	-4.711	Negative	5.356	212.150	4	2	58.200	0
2	α-Cubebene	Fail	Pass	0.052	Accepted	-4.408	Negative	18.693	204.190	0	0	0.000	0
3	β-Caryophyllone	Moderate	Pass	0.227	Accepted	-4.750	Negative	14.774	220.180	1	0	17.070	0
4	Phytosterols	Pass	Pass	0.013	Accepted	-4.756	Negative	16.686	414.390	1	1	20.230	0
5	UNII-0V56HXQ8N5	Moderate	Moderate	0.059	Accepted	-4.357	Negative	19.832	204.190	0	0	0.000	0
6	(-)-Alloaromadendrene	Pass	Pass	0.040	Accepted	-4.577	Negative	13.563	204.190	0	0	0.000	0
7	(-)-Camphene	Pass	Pass	0.077	Accepted	-4.463	Negative	9.346	136.130	0	0	0.000	0
8	(-)-*Cis*-Carveol	Fail	Pass	0.378	Accepted	-4.328	Negative	12.624	152.120	1	1	20.230	0
9	(-)-Linalool	Pass	Pass	0.493	Accepted	-4.375	Negative	9.738	154.140	1	1	20.230	0
10	(+)-α-phellandrene	Pass	Pass	0.617	Accepted	-4.383	Negative	12.660	136.130	0	0	0.000	0
11	(+)-δ-cadinene	Moderate	Moderate	0.051	Accepted	-4.469	Positive	7.421	204.190	0	0	0.000	0
12	(+)-Endo-β-bergamotene	Pass	Pass	0.063	Accepted	-4.466	Negative	16.946	204.190	0	0	0.000	0
13	(1S,2R,4S)-(-)-Bornyl acetate	Pass	Pass	0.243	Accepted	-4.552	Moderate	6.063	196.150	2	0	26.300	0
14	(1S)-1,7,7-Trimethylbicyclo[2.2.1]heptan-2-one	Pass	Pass	0.701	Accepted	-4.582	Negative	13.808	152.120	1	0	17.070	0
15	(E)-β-Farnesene	Fail	Fail	0.156	Accepted	-4.537	Moderate	13.186	204.190	0	0	0.000	0
16	(E)-α-bisabolene	Fail	Fail	0.092	Accepted	-4.502	Negative	17.581	204.190	0	0	0.000	0
17	(E)-β-Ocimene	Pass	Pass	0.678	Accepted	-4.434	Negative	14.171	136.130	0	0	0.000	0
18	1-Octen-3-ol	Fail	Fail	0.672	Accepted	-4.256	Negative	7.650	128.120	1	1	20.230	0
19	1S-α-Pinene	Pass	Pass	0.114	Accepted	-4.303	Negative	15.022	136.130	0	0	0.000	0
20	2,3-Dimethylaniline	Pass	Pass	0.583	Accepted	-4.255	Negative	10.496	121.090	1	2	26.020	0
21	2,5-Dimethoxybenzoic acid	Pass	Moderate	0.885	Accepted	-4.853	Positive	7.488	182.060	4	1	55.760	0
22	3-Carene	Pass	Pass	0.132	Accepted	-4.307	Negative	16.061	136.130	0	0	0.000	0
23	4-Terpineol	Pass	Pass	0.447	Accepted	-4.217	Negative	14.345	154.140	1	1	20.230	0
24	Acetic acid	Pass	Pass	0.791	Accepted	-5.218	Negative	1.609	60.020	2	1	37.300	0
25	Acetyleugenol	Pass	Pass	0.843	Accepted	-4.453	Moderate	8.457	206.090	3	0	35.530	0
26	α-Fenchene	Pass	Pass	0.099	Accepted	-4.460	Negative	10.559	136.130	0	0	0.000	0
27	α-Humulene	Fail	No	0.095	Accepted	-4.425	Negative	8.432	204.190	0	0	0.000	0
28	α-Terpineol	Pass	Pass	0.527	Accepted	-4.193	Negative	8.942	154.140	1	1	20.230	0
29	Apigenin	Fail	No	0.856	Accepted	-4.847	Positive	7.022	270.050	5	3	90.900	0
30	Apigenin 7-glucuronide	Fail	No	0.715	Rejected	-6.376	Positive	1.194	446.080	11	6	187.120	0
31	β-cadinene	Fail	Moderate	0.060	Accepted	-4.392	Negative	17.975	204.190	0	0	0.000	0
32	β-Carotene	Moderate	No	0.076	Rejected	-6.003	Negative	0.229	536.440	0	0	0.000	0
33	β-Caryophyllene	Pass	Pass	0.048	Accepted	-4.517	Negative	9.943	204.190	0	0	0.000	0
34	β-Pinene	Pass	Pass	0.107	Accepted	-4.460	Negative	10.097	136.130	0	0	0.000	0
35	*Bis*-acetic acid	Fail	No	0.998	Rejected	-7.722	Positive	12.869	1700.170	46	25	777.980	1 alert
36	Carotene	Pass	No	0.036	Rejected	-5.634	Negative	0.671	536.440	0	0	0.000	0
37	Carvacrol	Fail	Pass	0.671	Accepted	-4.436	Negative	11.335	150.100	1	1	20.230	1 alert
38	*Cis*-Anethole	Pass	Moderate	0.638	Accepted	-4.440	Negative	11.146	148.090	1	0	9.230	0
39	Decanal	Fail	Pass	0.456	Accepted	-4.551	Negative	5.049	156.150	1	0	17.070	0

40	Dehydro-p-cymene	Pass	Pass	0.568	Accepted	-4.344	Moderate	10.755	132.090	0	0	0.000	0
41	Dipentene	Fail	Pass	0.233	Accepted	-4.320	Negative	11.517	136.130	0	0	0.000	0
42	Estragole	Moderate	Moderate	0.577	Accepted	-4.308	Negative	12.054	148.090	1	0	9.230	0
43	Eucalyptol	Pass	Pass	0.352	Accepted	-4.414	Negative	8.066	154.140	1	0	9.230	0
44	Eugenol	Fail	Pass	0.887	Accepted	-4.373	Negative	14.042	164.080	2	1	29.460	0
45	γ-Selinene	Pass	Pass	0.088	Accepted	-4.577	Negative	13.350	204.190	0	0	0.000	0
46	Geranyl acetate	Pass	Pass	0.506	Accepted	-4.420	Moderate	9.707	196.150	2	0	26.300	0
47	Isoeugenol	Pass	Moderate	0.880	Accepted	-4.579	Negative	13.435	164.080	2	1	29.460	0
48	L-ascorbic acid	Fail	Fail	0.928	Accepted	-5.917	Positive	9.964	176.030	6	5	114.290	0
49	Linolenic acid	Fail	Fail	0.710	Accepted	-4.631	Negative	4.877	278.220	2	1	37.300	0
50	Luteolin-7-O-glucuronide	Fail	Fail	0.855	Rejected	-6.471	Positive	1.614	462.080	12	7	207.350	1 alert
51	Methyleugenol	Moderate	Pass	0.848	Accepted	-4.338	Negative	11.466	178.100	2	0	18.460	0
52	Molludistin	Fail	Fail	0.290	Accepted	-5.776	Positive	3.398	416.110	9	5	149.820	0
53	Myrcene	Pass	Pass	0.453	Accepted	-4.402	Moderate	13.108	136.130	0	0	0.000	0
54	Nerol	Fail	Pass	0.737	Accepted	-4.299	Positive	12.604	154.140	1	1	20.230	0
55	Octadeca-9,12-dienoic acid	Moderate	Fail	0.628	Accepted	-4.733	Negative	3.327	280.240	2	1	37.300	0
56	Octadecanoate	Fail	Fail	0.476	Accepted	-5.068	Negative	2.425	284.270	2	1	37.300	0
57	Oleic acid	Moderate	Fail	0.546	Accepted	-4.922	Negative	2.573	282.260	2	1	37.300	0
58	Orientin	Fail	Fail	0.724	Rejected	-6.208	Positive	5.042	448.100	11	8	201.280	1 alert
59	Palmitic acid	Fail	Fail	0.610	Accepted	-5.027	Negative	2.377	256.240	2	1	37.300	0
60	Thymol	Fail	Pass	0.682	Accepted	-4.387	Negative	9.444	150.100	1	1	20.230	0
61	Ursolic acid	Moderate	Pass	0.017	Accepted	-5.221	Negative	3.671	456.360	3	2	57.530	0

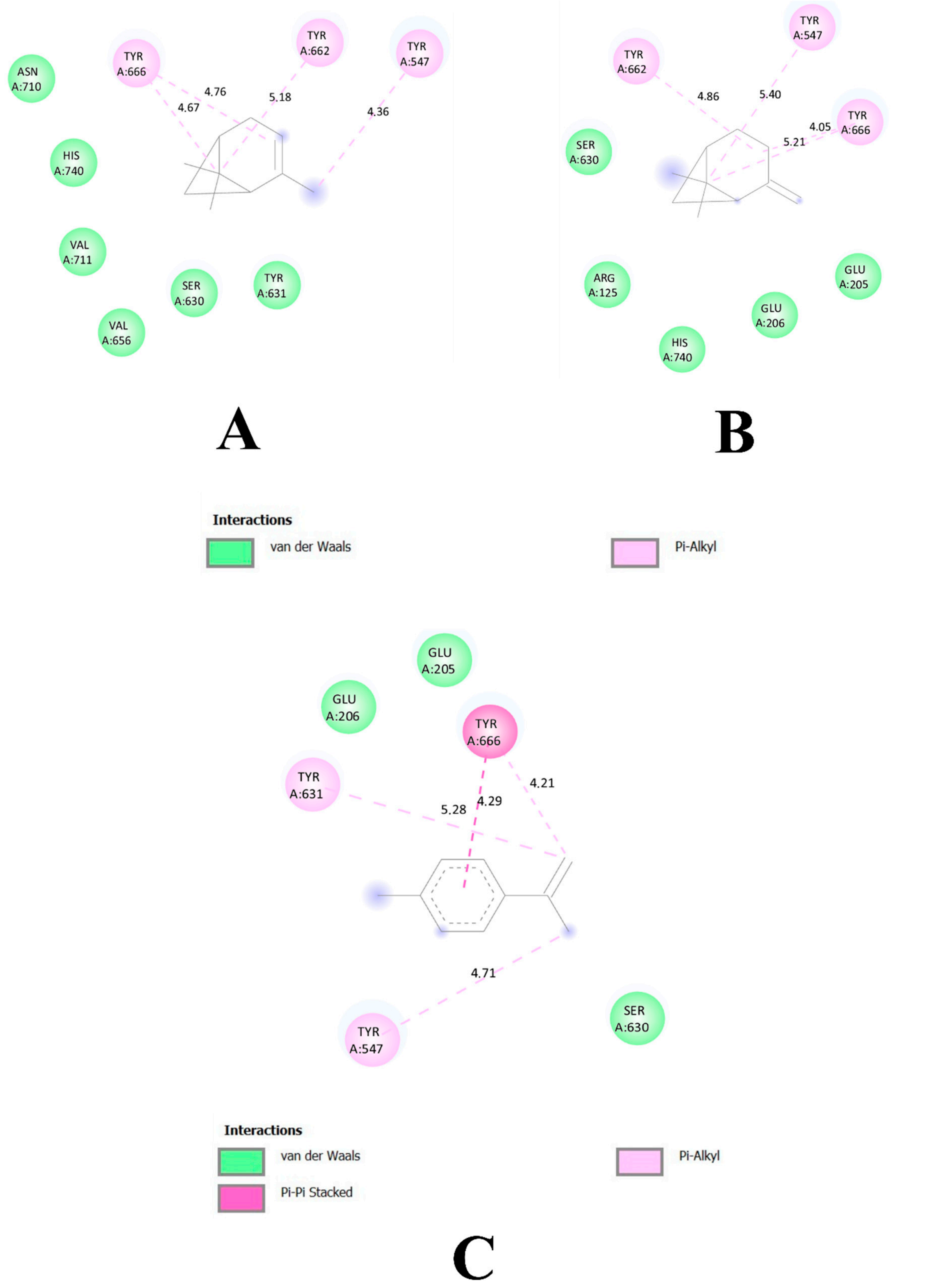

Supplementary Figure S1: Binding interactions of (A) 1S-α-pinene (binding affinity: -5.3 kcal/mol), (B) β-pinene (binding affinity: -5.2 kcal/mol), and (C) Dehydro-p-cymene (binding affinity: -5.5 kcal/mol) with DPP4 protein.

Supplementary Table S2: Comparative account of binding interactions during molecular docking and dynamics simulation studies conducted for experimental compounds.

Compounds	Binding interactions during molecular docking simulation	Binding interactions during molecular dynamics simulation
1S-α-pinene	Tyr666, Val656, Tyr631, Trp659, Tyr662	Tyr666, Tyr662, Phe357, Ser657, Tyr547,
β-pinene	Tyr666, Val656, Tyr631, Trp659, Tyr662	Tyr666, Tyr662, Ser 630, Asp663, Ser657, Tyr547
Dehydro-p-cymene	Tyr666, Val656, Val711, Phe357, Tyr631, Trp659, Tyr662	Tyr666, Trp659, Phe357, Ser657, Glu668, Ser630
Saxagliptin	Tyr547, Tyr662, Tyr666, Glu206	Tyr666, Tyr547, His740, Phe357, Ser630

Supplementary Table S3: Common binding interactions observed between molecular docking and dynamics simulation studies conducted for experimental compounds**.**

Compounds	Common binding interactions
1S-α-pinene	Tyr666 and Tyr662
β-pinene	Tyr666 and Tyr662
Dehydro-p-cymene	Tyr666 and Trp659
Saxagliptin	Tyr666 and Tyr547
